# Web-Based Data Collection for Older Adults Living With HIV in a Clinical Research Setting: Pilot Observational Study

**DOI:** 10.2196/18588

**Published:** 2020-11-11

**Authors:** Katherine Tassiopoulos, Carla Roberts-Toler, Carl J Fichtenbaum, Susan L Koletar

**Affiliations:** 1 Department of Epidemiology Harvard TH Chan School of Public Health Boston, MA United States; 2 Center for Biostatistics in AIDS Research Harvard TH Chan School of Public Health Boston, MA United States; 3 Division of Infectious Diseases Department of Internal Medicine University of Cincinnati College of Medicine Cincinnati, OH United States; 4 Division of Infectious Diseases Department of Medicine Ohio State University Columbus, OH United States

**Keywords:** HIV, aging, web-based data collection, longitudinal follow-up, mobile phone

## Abstract

**Background:**

Longitudinal follow-up of older persons living with HIV is essential for the ascertainment of aging-related clinical and behavioral outcomes, and self-administered questionnaires are necessary for collecting behavioral information in research involving persons living with HIV. Web-based self-reported data collection results in higher data quality than paper-and-pencil questionnaires in a wide range of populations. The option of remote web-based surveys may also increase retention in long-term research studies. However, the acceptability and feasibility of web-based data collection in clinical research involving older persons living with HIV have never been studied.

**Objective:**

This study aims to assess the acceptability and feasibility of a web-based survey to collect information on sexual, substance use, and physical activity behaviors; compare the data quality of the web-based survey with that of a paper-and-pencil questionnaire; and summarize web-based survey metrics.

**Methods:**

This pilot study took place within the AIDS Clinical Trials Group A5322 study, a longitudinal cohort of men and women living with HIV (aged ≥40 years), followed at 32 clinical sites in the United States and Puerto Rico. A total of 4 sites participated in this study. A web-based survey was created using self-administered questionnaires typically completed in A5322 via paper and pencil. Pilot study participants completed these questionnaires via web-based survey at one research visit in lieu of paper-and-pencil administration. Two questions were added to assess feasibility, defined as participants’ perception of the ease of web-based survey completion (very hard, hard, easy, very easy), and their preferred format (computer or tablet, paper and pencil, no preference) for completing the questions in the future (acceptability). Feasibility and acceptability were summarized overall and by demographic and clinical characteristics; the proportion of evaluable data by web-based survey versus previously administered paper-and-pencil questionnaires (data quality) was compared for each question.

**Results:**

Acceptability and feasibility were high overall: 50.0% (79/158) preferred computer or tablet, 38.0% (60/158) reported *no preference*, and 12.0% (19/158) preferred paper and pencil; 93.0% (147/158) reported survey completion easy or very easy. Older age was associated with lower odds of preferring computer or tablet to paper and pencil (odds ratio per 1-year increase in age: 0.91, 95% CI 0.85-0.98). Individuals who found the survey hard or very hard had a lower median neurocognitive test score than those who found it easy or very easy. Data quality with web-based survey administration was similar to or higher than that with paper-and-pencil administration for most questions.

**Conclusions:**

Web-based survey administration was acceptable and feasible in this cohort of older adults living with HIV, and data quality was high. Web-based surveys can be a useful tool for valid data collection and can potentially improve retention in long-term follow-up studies.

## Introduction

Maintaining sustained research participation is a critical challenge for longitudinal epidemiologic studies [1,2]. Factors that can negatively affect retention include study fatigue, work and personal commitments, and relocation away from the site of a research clinic. These factors may particularly affect studies designed to have extended follow-up durations. For older study participants and those with chronic health conditions, additional factors such as health limitations and difficulty traveling can make attendance at research study visits problematic [3].

Web-based data collection methods that allow the flexibility of remote survey completion may help improve retention in long-term studies [1]. These methods allow for data collection wherever a participant has access to a secure internet connection and ensure confidentiality of responses to potentially sensitive questions, and the embedding of skip patterns and out-of-range response checks and prompts can improve data quality. Data completeness has also been shown to be superior with web-based versus paper-and-pencil data collection [4], and data entry keying errors are reduced because this process is automated [5]. To achieve these data quality advantages, however, surveys must be perceived by study participants as acceptable and easy to complete. This is particularly true with older study participants, who may not be comfortable or familiar with web-based technologies. In 2017, 42% of US individuals aged ≥65 years owned a smartphone, with this proportion decreasing to 31% for those aged 75-79 years and 17% for those aged ≥80 years. In addition, 82% of those aged 65-69 years used the internet, a proportion similar to that of the overall population, but this decreased to 44% for those aged ≥80 years [6]. Particularly among older adults with certain disabilities or activity-limiting impairments, the use of email, texting, and the internet is lower than for those without these impairments [7]. Web-based technologies for research data collection have been used successfully with older adult study populations [8-11], and among older persons living with HIV, web-based and mobile apps have been evaluated for their usefulness and acceptability in health care settings [12-14]. However, to our knowledge, few studies have assessed the feasibility, acceptability, and data quality of web-based technologies for clinical research with older persons living with HIV [15], who may be living with physical and neurocognitive deficits because of their long-term HIV infection [16-20].

The AIDS Clinical Trials Group (ACTG) A5322 study is examining a wide range of clinical and behavioral end points in individuals aging with HIV. Data collection methods include interviews, physical examinations, and chart abstraction as well as self-administered questionnaires that are completed by hand (paper-and-pencil format). Once completed, these questionnaires are handed to research clinic staff in sealed envelopes and mailed to a data management center where responses are keyed. This process can result in incomplete, missed, or lost questionnaires. In addition, although these forms were designed for ease of completion, they do contain free-text responses as well as specific instructions, including those for skip patterns. Participants may have difficulty following the instructions, and the resulting data can be of poor quality because of issues such as skipping questions inappropriately and entering out-of-range free-text responses. Opportunities to clean these data are inherently limited, as data managers cannot query site staff or participants regarding their responses, and as a result, some information in these forms cannot be used.

We piloted a web-based survey that adapted 3 paper-and-pencil questionnaires administered in ACTG A5322. Our overall objective is to determine whether behavioral data collected via paper-and-pencil format could be successfully collected using web-based surveys. This was designed as the initial step of a longer-term goal of incorporating web-based surveys into the study’s regular schedule of evaluations, including potential expansion into remote data collection. Our specific aims are to (1) assess the acceptability and feasibility of the web-based survey and identify demographic and health characteristics associated with these measures; (2) compare the data quality of the web-based survey with that of the paper-and-pencil questionnaire; and (3) summarize web-based survey metrics, including frequency of and reasons for survey noncompletion and frequency of *rather not answer* responses.

## Methods

### Study Population

The A5322 study is an ongoing, long-term observational study following older men and women living with HIV for characterization and evaluation of age-related outcomes. Participants had previously been followed in ACTG A5001, another long-term observational study of participants who had received either their initial HIV antiretroviral treatment medication (treatment naive) or a salvage therapy through an ACTG randomized clinical trial. When the A5001 follow-up ended, participants who had been treatment naive at the time of enrollment in their initial ACTG clinical trial and were aged ≥40 years were eligible to enroll in the A5322 study. Altogether, 1035 participants were enrolled between November 2013 and July 2014 at 32 clinical research sites across the United States, including Puerto Rico. Participants were previously evaluated semiannually (now annually) for immunologic, virologic, and clinical parameters and annually for behavioral parameters. All participants provided written informed consent before enrollment into A5322, and the study was approved by the local institutional review board at each site.

### Study Design

A total of 4 sites were chosen for this pilot study based on the number of participants they had enrolled into A5322, the proportion of participants who spoke English as their first language, and the availability of a laptop or desktop with wired internet and access to a private space in which to complete the survey. All participants at the 4 sites whose primary language was English were eligible to participate in the pilot study. The web-based survey was administered during a single visit, which took place at the 4 sites, in place of the paper-and-pencil questionnaires typically administered.

### Survey Development

The survey was developed using Illume, a commercial software tool designed by DatStat (DatStat, Incorporated). The survey was developed only in English and consisted of 3 questionnaires on recent sexual behaviors, current and past substance use, and physical activity (the latter using the International Physical Activity Questionnaire Short Form [21]). At the end of the survey, we included 2 questions to assess acceptability and feasibility (perceived ease of completion). Automatic skip patterns and prompts for participants to re-enter responses that were out-of-range were included. All questions were worded to match the phrasing of the 3 paper-and-pencil questionnaires, except when modifications were necessary to accommodate embedded skip patterns. All questions, except for acceptability and feasibility, included *rather not answer* response options so that participants would not be forced to answer questions and to make preferences for not answering questions transparent.

### Outcomes

Frequency of and reasons for noncompletion of the survey were collected on a tracking form completed by the clinic staff. Acceptability was assessed with a three-category variable from the question, “In the future, I would prefer to complete the survey by *computer/tablet, paper/pencil, no preference*.” Feasibility was assessed with a four-category variable (*very hard, hard, easy,* and *very easy*).

The data quality of the web-based survey and paper-and-pencil responses was assessed by comparing the proportion of evaluable responses to each question in the web-based survey with that obtained from previously administered paper-and-pencil questionnaires. Evaluable data were defined as any valid response, excluding *rather not answer*. Nonevaluable data were defined as missing, *rather not answer* responses, and out-of-range responses for free-text questions. An example of an out-of-range free-text response would be writing *30* in response to the question, “In the past 7 days, on how many days did you do vigorous exercise?” Logically missing data that resulted from appropriate responses to prior questions were not considered missing (eg, if no vigorous exercise was reported, questions on the number of days and time spent doing vigorous exercise were skipped).

### Covariates

Demographic and functional fitness characteristics were assessed at the visit closest to the survey administration unless otherwise indicated. The variables included age, race/ethnicity, sex, education (assessed during A5001 follow-up), history of comorbidities (diabetes, kidney disease, liver disease, cardiovascular disease, stroke, hepatitis C–positive serology, and cancer [within 5 years]), frailty, disability; and neurocognitive function and impairment. Frailty was assessed using the Fried Frailty assessment, which includes 4-m walk speed; grip strength; and self-reported unintentional weight loss, exhaustion, and low activity [22]. Individuals meeting 3 to 5 components are categorized as frail, those meeting 1 to 2 components are categorized as prefrail, and those meeting 0 components are categorized as nonfrail. For this analysis, we used a two-category frailty variable (frail vs nonfrail/prefrail). Disability was assessed with the Lawton-Brody Instrumental Activities of Daily Living (IADL) questionnaire using self-reported limitations in performing 8 tasks: housekeeping, money management, cooking, transportation, telephone use, shopping, laundry, and medication management [23]. We defined IADL disability as ≥1 limitation. We assessed neurocognitive function using the Trail-Making Tests A and B and the Wechsler Adult Intelligence Scale-Revised Digit Symbol subtest. The raw scores from these 3 evaluations were standardized by age, sex, race/ethnicity, and education and combined into one summary z-score (NPZ-3 score). Neurocognitive impairment was defined as having at least one z-score ≥2 SDs below the mean or at least two z-scores ≥1 SD below the mean. Raw scores were normalized only for participants who were Black, White, or Hispanic.

### Statistical Analysis

#### Survey Metrics

To compare the frequency of *rather not answer* responses by acceptability and feasibility, the total number of *rather not answer* responses given by each participant across all survey questions was summed, and this summed value was then dichotomized to 0 or ≥1 *rather not answer* responses. Chi-square tests were used for comparisons.

#### Acceptability and Feasibility

We compared the distribution of acceptability by demographic and health characteristics using chi-square tests for categorical variables and the Kruskal-Wallis test for continuous variables. Multinomial logistic regression models were fit to evaluate the association of these variables with acceptability as a three-category variable, *paper and pencil* as the reference category in one model, and *no preference* as the reference in the second model. Univariate models were first fit with each individual variable. All variables with a *P* value of less than .10 in the univariable model were then included in a multivariable model. Feasibility was compared by age and neurocognitive score using the Wilcoxon test. As only a few individuals reported that the survey was hard or very hard, feasibility was evaluated as a dichotomous variable (easy/very easy vs hard/very hard).

#### Data Quality

We compared the proportion of evaluable responses to each question by survey format using chi-square tests. The first comparison was between pilot study participants’ web-based responses and their responses to the most recently completed paper-and-pencil questionnaire. A second comparison was between pilot study participants’ web-based responses and responses of all A5322 participants on their most recently completed paper-and-pencil questionnaire. Chi-square tests were used for these comparisons instead of a matched approach because the number of responses to many of the questions differed by mode of administration. Therefore, it was not possible to use a matched approach that would take into account within-person correlations.

Finally, we compared the proportion of evaluable data for all questions within the web-based survey by age and neurocognitive impairment.

SAS 9.4 was used for all analyses (SAS Institute).

## Results

### Survey Metrics

A total of 180 participants at the 4 sites were eligible to participate in the pilot study. Of these 180 participants, 159 (88.3%) completed the web-based survey; for the 21 eligible participants who did not complete the survey, the following reasons were provided: participant declined (8/180, 4.4%), clinic error (7/180, 3.9%), technical difficulties (3/180, 1.7%), time constraints (2/180, 1.1%), and cognitive impairment (1/180, 0.6%).

Of the 35 questions in the web-based survey that included a *rather not answer* response option, 18 questions (51%) received at least one (range 1-4) *rather not answer* response. Multimedia Appendix 1 lists the questions that had one or more *rather not answer* responses. Of 159 participants, 24 (15.1%) responded *rather not answer* to one or more of the survey questions, with 16 responding *rather not answer* to 1 question, 6 responding to 2 questions, and 2 responding to 3 questions.

### Acceptability and Feasibility

Table 1 summarizes the participant characteristics, overall and by acceptability. Most participants were men, and the median age was 54 years (IQR 49-61). Most participants had more than a high school education, and the majority reported no IADL limitations.

**Table 1:** Demographic and functional fitness by acceptability.

Characteristic		Prefer computer/tablet (n=79)	Prefer paper-and-pencil questionnaire (n=19)	No preference (n=60)	Total (N=158^a^)	*P* value
Age (years), median (Q1-Q3)		53 (48 to 60)	59 (52 to 66)	55 (50 to 61.5)	54 (49 to 61)	.04^b^
Sex, male^c^, n (%)		62 (79)	14 (74)	53 (88)	129 (81.7)	.21^d^
**Race and ethnicity, n (%)**						.75^d^
	White, non-Hispanic	43 (54)	13 (68)	39 (65)	95 (60.1)	
	Black, non-Hispanic	30 (38)	6 (32)	19 (32)	55 (34.8)	
	Hispanic (regardless of race)	3 (4)	0 (0)	2 (3)	5 (3.2)	
	Asian, Pacific Islander	2 (3)	0 (0)	0 (0)	2 (1.3)	
	More than one race	1 (1)	0 (0)	0 (0)	1 (0.6)	
NPZ-3 score: median (Q1-Q3)^e^		0.55 (−0.20 to 1.20)	0.50 (−0.20 to 1.20)	0.75 (0.10 to 1.25)	0.60 (−0.10 to 0.20)	.34^b^
Neurocognitive impairment^e,f^, n (%)		9 (11)	2 (11)	4 (7)	15 (9.5)	.59^d^
Greater than high school education level^f^, n (%)		48 (61)	12 (63)	43 (72)	103 (65.2)	.55^d^
Frail, n (%)		8 (10)	1 (5)	5 (8)	14 (8.9)	.79^d^
≥1 IADL^g^ limitation^f^, n (%)		19 (24)	2 (11)	4 (7)	25 (15.8)	.02^d^
History of any comorbidity, n (%)		68 (86)	16 (84)	52 (87)	136 (86.1)	.96^d^

^a^One participant (Black non-Hispanic male, high school education, no IADL limitations) skipped the acceptability question.

^b^Kruskal-Wallis test.

^c^Sex at birth. Information on gender not available.

^d^Chi-square test.

^e^Black, White, and Hispanic participants only.

^f^The following variables had missing observations: neurocognitive impairment (N=3, all chose computer/tablet), education (N=3, all chose computer/tablet), and IADL limitations (N=1, chose *paper and pencil*, and N=2, *no preference*).

^g^IADL: Instrumental Activities of Daily Living.

Overall, 50.0% (79/158) of participants indicated that they would in the future prefer to answer the questionnaires via computer or tablet, 12.0% (19/158) said they would prefer the *paper-and-pencil* format, and 38.0% (60/158) had *no preference*. The median (IQR) age of participants who preferred the *paper-and-pencil* questionnaire was 59 years (IQR 52-56), compared with 53 years (IQR 48-60) for those who preferred computer or tablet and 55 years (IQR 50-61.5) for those who indicated *no preference*. Those with ≥1 IADL limitation were more likely to prefer the computer or tablet option (19/25, 76%) than either *paper and pencil* (2/25, 8%) or *no preference* (4/25, 16%), whereas those with no self-reported limitations were similarly as likely to prefer computer or tablet (60/131, 45.8%) or have *no preference* (55/131, 42.0%). There were no differences in acceptability by other characteristics.

In an adjusted multinomial logistic regression model including age and IADL, with *paper and pencil* as the reference group, older age was associated with lower odds of preferring the computer or tablet format (odds ratio [OR] per 1-year increase in age 0.91, 95% CI 0.85-0.98; Table 2). With *no preference* as the reference group, those with ≥1 IADL were more likely to prefer the computer or tablet (OR 4.42, 95% CI 1.40-13.9; Table 3).

**Table 2:** Multinomial logistic regression model: factors associated with acceptability (paper and pencil as the reference group).

Variable and outcome		OR^a^ (95% CI)	*P* value
**Age at survey completion, years**			
	Computer/tablet	0.91 (0.85-0.98)	.01
	No preference	0.95 (0.89-1.02)	.14
**IADL^b^ impairment (≥1 impaired categories)**			
	Computer/tablet	2.72 (0.55-13.5)	.22
	No preference	0.62 (0.1-3.74)	.60

^a^OR: odds ratio.

^b^IADL: Instrumental Activities of Daily Living.

**Table 3:** Multinomial logistic regression model: factors associated with acceptability (no preference as the reference group).

Variable and outcome		OR^a^ (95% CI)	*P* value
**Age at survey completion, years**			
	Computer/tablet	0.96 (0.92-1.01)	.11
	Paper-and-pencil questionnaire	1.05 (0.98-1.13)	.14
**IADL^b^ impairment (≥1 impaired categories)**			
	Computer/tablet	4.42 (1.4-13.9)	.01
	Paper-and-pencil questionnaire	1.62 (0.27-9.87)	.60

^a^OR: odds ratio.

^b^IADL: Instrumental Activities of Daily Living.

Most participants (147/158, 93.0%) reported that the web-based survey was easy or very easy to complete. Individuals who found the survey hard or very hard had a lower median NPZ-3 score than did those who found it easy or very easy; age was not associated with feasibility (Table 4).

**Table 4:** Age and NPZ-3 score by feasibility.

Characteristics		Hard/very hard (n=10)	Easy/very easy (n=148)	Total (N=158^a^)	*P* value^b^
**NPZ-3 score**					
	Minimum to maximum	−1.10 to 3.00	−2.00 to 3.20	−2.00 to 3.20	
	Median (Q1-Q3)	−0.30 (−1.00 to 0.20)	0.70 (0.00 to 1.20)	0.60 (−0.10 to 1.20)	.02
**Age at survey completion**					
	Minimum to maximum	44 to 77	44 to 79	44 to 79	
	Median (Q1 to Q3)	49.5 (48.0 to 52.0)	54.5 (49.5 to 61.0)	54.0 (49.0 to 61.0)	.16

^a^One participant skipped the feasibility question.

^b^Wilcoxon test.

Participants who thought the web-based survey was easy or very easy to complete were more likely to prefer to answer future questions via computer or tablet than those who found it hard or very hard (*data not shown*). There were no differences in acceptability or feasibility between participants with ≥1 versus no *rather not answer* responses (Multimedia Appendix 2).

### Data Quality

Comparisons of evaluable responses by questionnaire administration are summarized in Table 5. Although overall data quality was high for both formats, for most questions, the web-based survey yielded a proportion of evaluable data that was similar to or greater than for the paper-and-pencil questionnaire. Data quality was higher with the web-based survey for most of the sexual behavior questions and all the physical activity questions; the greatest difference in the latter was for questions about the length of time performing various physical activities. The only 2 questions for which data quality was low in both formats were anal sex with women with condom and oral sex with women with condom. These were endorsed by a few individuals (N=14, web-based survey; N=8, *paper-and-pencil* questionnaire) such that the number of missed responses resulted in very low proportions of evaluable responses. Almost all substance use behavior responses were similarly evaluable in both formats. The results were similar when comparing the responses of those who completed the web-based survey with the paper-and-pencil responses of all A5322 participants (Multimedia Appendix 3). We found no differences in data quality when we examined the proportion of evaluable data *within* the web-based survey by age and neurocognitive impairment (*data not shown*).

**Table 5:** Evaluable responses by questionnaire format (N=159).

Question		Paper and pencil: proportion of evaluable responses		Web-based survey: proportion of evaluable responses		*P* value
		n (%)	N	n (%)	N	
**Sexual behavior**						
	Any sexual partners	140 (88.1)	159	155 (97.5)	159	.001
	Number of sexual partners	67 (73)	92	75 (90)	83	.003
	Oral sex with a man	81 (88)	92	65 (71)	92	.004
	Oral sex with a man with condom	59 (82)	72	57 (90)	63	.15
	Oral sex with a woman	73 (79)	92	77 (93)	83	.01
	Oral sex with a woman with condom	8 (28)	29	7 (54)	13	.10
	Vaginal sex	75 (82)	92	78 (94)	83	.01
	Vaginal sex with condom	22 (54)	41	23 (79)	29	.03
	Anal sex with a man	76 (83)	92	79 (95)	83	.009
	Anal sex with a man with condom	40 (69)	58	41 (91)	45	.007
	Anal sex with a woman	69 (75)	92	79 (95)	83	<.001
	Anal sex with a woman with condom	1 (4)	24	3 (43)	7	.007
	Any new sexual partners	83 (90)	92	79 (95)	83	.21
	Number of partners who know your HIV status	84 (91)	92	75 (90)	83	.83
	Number of partners with known HIV status	86 (93)	92	77 (93)	83	.85
**Substance use**						
	How often drink alcohol	159 (100.0)	159	158 (99.4)	159	.32
	How many drinks containing alcohol	99 (98.0)	101	96 (96.0)	100	.40
	How often binge drink alcohol	101 (100.0)	101	99 (99.0)	100	.31
	Not getting things done because of alcohol	98 (97.0)	101	99 (99.0)	100	.32
	Emotional problems from alcohol	96 (95.0)	101	100 (100.0)	100	.02
	Last time used tobacco	156 (98.1)	159	157 (98.7)	159	.65
	Last time used marijuana	157 (98.7)	159	158 (99.4)	159	.56
	Last time used cocaine	157 (98.7)	159	158 (99.4)	159	.56
	Last time used heroin	156 (98.1)	159	159 (100.0)	159	.08
	Last time used amphetamines	154 (96.9)	159	159 (100.0)	159	.02
	Last time used other nonprescribed substance	152 (95.6)	159	159 (100.0)	159	.007
	Not getting things done because of substance use	86 (97)	89	98 (98.0)	100	.56
	Emotional problems from substance use	85 (96)	89	98 (98.0)	100	.33
**Physical activity**						
	How many days spent doing vigorous activities	153 (96.2)	159	159 (100.0)	159	.01
	How much time spent doing vigorous activities	79 (90)	88	85 (98)	87	.03
	How many days spent doing moderate activities	149 (93.7)	159	159 (100.0)	159	.001
	How much time spent doing moderate activities	91 (82.7)	110	102 (99.0)	103	<.001
	How many days spent walking ≥10 min	148 (93.1)	159	159 (100.0)	159	<.001
	How much time spent walking	119 (87.5)	136	140 (99.3)	141	<.001
	How much time spent sitting	126 (79.2)	159	156 (98.1)	159	<.001

## Discussion

### Principal Findings

To our knowledge, this is one of the first studies on the feasibility and acceptability of web-based data collection for clinical research purposes among older adults living with HIV, and our findings indicate that web-based surveys can successfully be implemented in research with this population. The perceived acceptability and feasibility of the web-based survey were high, and almost all participants found the survey to be easy or very easy to complete. The data quality of responses via the web-based survey was similar to or higher than that for the same questions in the paper-and-pencil questionnaires.

Although overall acceptability and feasibility were high, there were differences by demographic and clinical factors that need to be taken into account when deciding how to incorporate web-based data collection. Younger adults were more likely than older adults to prefer a web-based format. Although the adoption of web-based technologies has steadily increased in recent years among older adults in the United States, it continues to lag behind those of younger adults, as does confidence in one’s ability to use these technologies [6]. Although age was not associated with the feasibility of web-based survey completion, neurocognitive function was; participants with lower neurocognitive test scores were less likely to report that the survey was easy or very easy to complete.

Although data quality within the web-based format was not affected by either age or neurocognitive function, the fact that participant preference did depend on these factors has to be considered when determining how to incorporate web-based surveys in a clinical research study. A mixed mode study design is likely needed, with participants given the option of either web-based or paper-and-pencil administration [24]. This option will become increasingly important, as participants continue to age and perhaps experience impairments in neurocognitive function. A growing body of literature indicates the equivalence of paper and pencil and electronic methods of patient-reported outcomes, particularly when care has been taken to minimize the differences between modes with respect to wording, interpretation, and response options [25,26]. Providing choice via a mixed mode study design has been shown to increase motivation among some participants [27] as well as lead to higher response rates [28] and a more representative study sample makeup [29].

Web-based survey acceptability was not affected by the presence of frailty or health comorbidities. Frailty was also not associated with preference or interest in technology use in a previous study of older adults [30]. Indeed, we observed that participants who reported one or more limitations in IADL were much more likely to prefer completing the questions in the future using a computer or tablet than to report *no preference*. A study that evaluated older adults’ use of technology for personal or health-related tasks found a higher prevalence of use among those with certain health issues (pain and breathing problems) that limited activities of daily living. They concluded that technology might be useful to enhance communication and completion of tasks by removing some barriers associated with completing these tasks offline [7]. However, other research on older adults who report not using or discontinuing the use of web-based technologies cite functional impairments such as arthritis and visual deficits as reasons for nonuse [6,31]. Although future research is needed, it appears that web-based technology may facilitate research data collection for older adults with specific health-related limitations.

The findings of this pilot study indirectly inform a longer-term goal of incorporating remotely completed web-based assessments into A5322. In addition to age- or cognitive-based limitations with completing web-based assessments, remote completion also requires internet/smartphone access at home as well as the skills needed to independently access and submit web-based surveys. Given these caveats, the option of remote data completion may increase retention among participants who find it difficult to maintain regular clinic visits because of time, travel, or health restrictions. Although studies that rely solely on remote data collection are likely to have high attrition rates [32,33], a study design that incorporates remote web-based data collection while retaining the option of in-person study visits has been suggested as a method that can improve long-term retention [1].

### Limitations

This pilot study was limited to participants whose primary language was English, and the design required that the web-based questionnaires be completed on site. Therefore, the results might not be generalizable to non–English-speaking individuals or to those without adequate computer or tablet or internet access. We were unable to have participants complete the web-based and paper-and-pencil questionnaires at the same time and therefore were not able to compare frequencies of behaviors by format because we could not assume that the frequency of these behaviors would be stable over time. We did not take within-person correlations into account when comparing the proportion of evaluable responses across survey administration types. Many of the questions were designed to be answered only by participants who endorsed a leading question that triggered another question (eg, the leading question “in the past 6 months, have you had sex with another person?” would trigger subsequent questions on the type of partners and condom use). The number of participants answering each question differed by mode of administration because they were asked at different points in time, with different frequencies of behaviors reported. Therefore, a matched approach was not possible for all comparisons. However, we were able to use McNemar test for a subset of questions (those asked of all participants) to account for within-person correlations. In these situations, the McNemar *P* values were consistent with the chi-square *P* values (Multimedia Appendix 4).

### Conclusions

We found that in a group of older adults living with HIV being followed in a longitudinal observational study, completion of a web-based survey of questionnaires assessing sexual, substance use, and physical activity behaviors was perceived to be highly acceptable and feasible, with data quality on average being higher with the web-based versus paper-and-pencil format. As persons living with HIV continue to age, often with comorbidities and disabilities, long-term participation in research will become more challenging, even as the need to understand their health-related outcomes continues to grow [34]. Web-based technologies can be a useful tool for valid data collection and may be a way to optimize the retention of these individuals.

## Appendix

Multimedia Appendix 1 Web-based survey questions with ≥1 rather not answer response.

Multimedia Appendix 2 Rather not answer responses by acceptability and feasibility.

Multimedia Appendix 3 Evaluable responses by questionnaire format (comparing web-based surveys with paper-and-pencil questionnaires from all A5322 participants).

Multimedia Appendix 4 Comparison of evaluable responses—chi-square test versus McNemar test.

## Abbreviations

ACTG: AIDS Clinical Trials Group
IADL: Instrumental Activities of Daily Living
NIH: National Institutes of Health
OR: odds ratio
