# Supplementary material for: Web-Based Data Collection for Older Adults Living With HIV in a Clinical Research Setting: Pilot Observational Study
Source: J Med Internet Res. 2020 Nov 11;22(11):e18588. doi: 10.2196/18588 (PMC7688395; doi:10.2196/18588)
Supplement: Multimedia Appendix 1 [file jmir_v22i11e18588_app1.docx]

**Multimedia Appendix 1. Web-based Survey Questions with ≥ 1 Rather Not Answer (RNA) Responses**

| **Survey Question** | **Number of RNA Responses** |
| --- | --- |
| How many drinks containing alcohol | 4 |
| Any sexual partners | 4 |
| Number of sexual partners | 4 |
| How much time spent sitting | 3 |
| How much time spent on vigorous activities | 2 |
| Oral sex with a man | 2 |
| Oral sex with a woman | 2 |
| Last time used tobacco | 2 |
| Number of sex partners with known HIV status | 2 |
| How much time spent on moderate activities | 1 |
| How much time spent walking | 1 |
| How often drink alcohol | 1 |
| How often binge drink alcohol | 1 |
| Not getting things done because of alcohol | 1 |
| Last time used marijuana | 1 |
| Last time used cocaine | 1 |
| Vaginal sex | 1 |
| Vaginal sex with condom | 1 |

*Survey questions not listed had no RNA responses.*
