# Supplementary material for: Web-Based Data Collection for Older Adults Living With HIV in a Clinical Research Setting: Pilot Observational Study
Source: J Med Internet Res. 2020 Nov 11;22(11):e18588. doi: 10.2196/18588 (PMC7688395; doi:10.2196/18588)
Supplement: Multimedia Appendix 2 [file jmir_v22i11e18588_app2.docx]

**Multimedia Appendix 2: Rather Not Answer Responses by Acceptability and Feasibility**

|  | | **Number RNA responses** | |  | |
| --- | --- | --- | --- | --- | --- |
| **Characteristic** |  | **0 (N=135)** | **≥1 (N=24)** | **Total (N=159)** | ***P-Value**** |
| Future preference to answer questions | Computer/tablet | 67 (49.6%) | 12 (50%) | 79 (49.7%) | 0.59 |
|  | Paper/pencil questionnaire | 15 (11.1%) | 4 (17%) | 19 (12.0%) |  |
|  | No preference | 53 (39.3%) | 7 (29%) | 60 (37.7%) |  |
|  | Missing | 0 (0.0%) | 1 (4%) | 1 (0.6%) |  |
| Perceived ease | Hard | 8 (5.9%) | 2 (8%) | 10 (6.3%) | 0.61 |
|  | Easy | 127 (94.1%) | 21 (88%) | 148 (93.1%) |  |
|  | Missing | 0 (0.0%) | 1 (4%) | 1 (0.6%) |  |
| *Chi-Square Test | | | | | |
