# Supplementary material for: Web-Based Data Collection for Older Adults Living With HIV in a Clinical Research Setting: Pilot Observational Study
Source: J Med Internet Res. 2020 Nov 11;22(11):e18588. doi: 10.2196/18588 (PMC7688395; doi:10.2196/18588)
Supplement: Multimedia Appendix 3 [file jmir_v22i11e18588_app3.docx]

**Multimedia Appendix 3: Evaluable Responses by Questionnaire Format**

| **Question** | **Paper/Pencil*: Proportion of Evaluable Responses**  **(N=1035)** | **Web-based:**  **Proportion of Evaluable Responses**  **(N=159)** | ***P-value*** |
| --- | --- | --- | --- |
| ***Sexual Behavior***  Any sexual partners | 0.85 (876/1034) | 0.97 (155/159) | <0.001 |
| Number of sexual partners | 0.70 (448/637) | 0.90 (75/83) | <0.001 |
| Oral sex with a man | 0.81 (515/637) | 0.71 (65/92) | 0.023 |
| Oral sex with a man with condom | 0.72 (364/507) | 0.90 (57/63) | 0.001 |
| Oral sex with a woman | 0.72 (457/637) | 0.93 (77/83) | <0.001 |
| Oral sex with a woman with condom | 0.23 (54/240) | 0.54 (7/13) | 0.010 |
| Vaginal sex | 0.73 (468/637) | 0.94 (78/83) | <0.001 |
| Vaginal sex with condom | 0.41 (127/312) | 0.79 (23/29) | <0.001 |
| Anal sex with a man | 0.77 (492/637) | 0.95 (79/83) | <0.001 |
| Anal sex with a man with condom | 0.63 (270/427) | 0.91 (41/45) | <0.001 |
| Anal sex with a woman | 0.69 (439/637) | 0.95 (79/83) | <0.001 |
| Anal sex with a woman with condom | 0.03 (7/205) | 0.43 (3/7) | <0.001 |
| Any new sexual partners | 0.79 (501/637) | 0.95 (79/83) | <0.001 |
| Number of partners who know your HIV status | 0.83 (527/637) | 0.90 (75/83) | 0.08 |
| Number of partners with known HIV status | 0.83 (531/637) | 0.93 (77/83) | 0.026 |
|  |  |  |  |
| ***Substance Use*** |  |  |  |
| How often drink alcohol | 1.00 (1009/1011) | 0.99 (158/159) | 0.32 |
| How many drinks containing alcohol | 0.98 (607/620) | 0.96 (96/100) | 0.24 |
| How often binge drink alcohol | 0.99 (614/620) | 0.99 (99/100) | 0.98 |
| Not getting things done because of alcohol | 0.98 (607/620) | 0.99 (99/100) | 0.46 |
| Emotional problems from alcohol | 0.96 (594/620) | 1.00 (100/100) | 0.037 |
| Last time used tobacco | 0.99 (998/1011) | 0.99 (157/159) | 0.98 |
| Last time used marijuana | 0.97 (984/1011) | 0.99 (158/159) | 0.12 |
| Last time used cocaine | 0.97 (976/1011) | 0.99 (158/159) | 0.054 |
| Last time used heroin | 0.97 (979/1011) | 1.00 (159/159) | 0.023 |
| Last time used amphetamines | 0.97 (981/1011) | 1.00 (159/159) | 0.028 |
| Last time used other non-prescribed substance | 0.95 (956/1011) | 1.00 (159/159) | 0.003 |
| Not getting things done because of substance use | 0.95 (537/565) | 0.98 (98/100) | 0.19 |
| Emotional problems from substance use | 0.95 (535/565) | 0.98 (98/100) | 0.15 |
|  |  |  |  |
| ***Physical Activity*** |  |  |  |
| How many days spent doing vigorous activities | 0.87 (902/1034) | 1.00 (159/159) | <0.001 |
| How much time spent doing vigorous activities | 0.75 (440/587) | 0.98 (85/87) | <0.001 |
| How many days spent doing moderate activities | 0.87 (897/1034) | 1.00 (159/159) | <0.001 |
| How much time spent doing moderate activities | 0.75 (529/707) | 0.99 (102/103) | <0.001 |
| How many days spent walking ≥10 minutes | 0.86 (885/1034) | 1.00 (159/159) | <0.001 |
| How much time spent walking | 0.79 (723/916) | 0.99 (140/141) | <0.001 |
| How much time spent sitting | 0.77 (793/1034) | 0.98 (156/159) | <0.001 |

*Most recent paper/pencil survey from all A5322 participants.
