# Supplementary material for: Web-Based Data Collection for Older Adults Living With HIV in a Clinical Research Setting: Pilot Observational Study
Source: J Med Internet Res. 2020 Nov 11;22(11):e18588. doi: 10.2196/18588 (PMC7688395; doi:10.2196/18588)
Supplement: Multimedia Appendix 4 [file jmir_v22i11e18588_app4.docx]

| **Multimedia Appendix 4: Comparison of Evaluable Responses – Chi-square Test vs McNemar’s Test** | | | | |
| --- | --- | --- | --- | --- |
| **Question** | **Paper/Pencil: Proportion of Evaluable Responses** | **Web-based: Proportion of Evaluable Responses** | **p-value from**  **chi-square test** | **p-value from McNemar’s test** |
| ***Sexual Behavior*** |  |  |  |  |
| Any sexual partners | 0.88 (140/159) | 0.97 (155/159) | .001 | 0.0026 |
| ***Substance Use*** |  |  |  |  |
| How often drink alcohol | 1.00 (159/159) | 0.99 (158/159) | .32 | .32 |
| Last time used tobacco | 0.98 (156/159) | 0.99 (157/159) | .65 | 1 |
| Last time use marijuana | 0.99 (157/159) | 0.99 (158/159) | .56 | 1 |
| Last time used cocaine | 0.99 (157/159) | 0.99 (158/159) | .56 | 1 |
| Last time used heroin | 0.98 (156/159) | 1.00 (159/159) | .08 | .25 |
| Last time used amphetamines | 0.97 (154/159) | 1.00 (159/159) | .02 | .06 |
| Last time used other non-prescribed substance | 0.96 (152/159) | 1.00 (159/159) | .007 | .02 |
| ***Physical activity*** |  |  |  |  |
| How many days spent doing vigorous activities | 0.96 (153/159) | 1.00 (159/159) | .01 | .03 |
| How many days spent doing moderate activities | 0.94 (149/159) | 1.00 (159/159) | .001 | .002 |
| How many days spent walking ≥ 10 minutes | 0.93 (148/159) | 1.00 (159/159) | <.001 | .001 |
| How much time spent sitting | 0.79 (126/159) | 0.98 (156/159) | <.001 | .002 |
| Comparison of chi-square and McNemar’s tests for subset of questions designed to be answered by all participants. | | | | |
